# Lack of Association between *Chlamydophila psittaci* and Ocular Adnexal MALT Lymphoma in Korean Patients—Is the Geographic or Genetic Difference Significant?

**DOI:** 10.3390/diagnostics11112069

**Published:** 2021-11-09

**Authors:** Uiju Cho, Inju Cho, Sung Hak Lee, Suk Woo Yang, Seok Goo Cho, Youn Soo Lee, Hye Won Lee, Gyeongsin Park

**Affiliations:** 1 Department of Pathology, St. Vincent’s Hospital, College of Medicine, The Catholic University of Korea, Seoul 06591, Korea; hailtoya@catholic.ac.kr; 2 Department of Pathology, Eunpyeong St. Mary’s Hospital, College of Medicine, The Catholic University of Korea, Seoul 06591, Korea; injucho81@gmail.com; 3 Department of Pathology, St. Mary’s Hospital, College of Medicine, The Catholic University of Korea, Seoul 06591, Korea; hakjjang@catholic.ac.kr (S.H.L.); lys9908@catholic.ac.kr (Y.S.L.); 4 Department of Opthalmology, St. Mary’s Hospital, College of Medicine, The Catholic University of Korea, Seoul 06591, Korea; yswoph@catholic.ac.kr; 5 Department of Internal Medicine, St. Mary’s Hospital, College of Medicine, The Catholic University of Korea, Seoul 06591, Korea; chosg@catholic.ac.kr; 6 Department of Pathology, School of Medicine, Keimyung University, Daegu 42601, Korea; 7 Cancer Research Institute, The Catholic University of Korea, Seoul 06591, Korea

**Keywords:** *Chlamydophila* *psittaci*, *Chlamydia*, lymphoma, B cell, marginal zone, MALT lymphoma, eye neoplasms

## Abstract

*Clamydophila psittaci* (*C. psittaci*) has been proposed to be an etiologic factor in extranodal marginal zone lymphoma of mucosa-associated lymphoid tissue (MALT lymphoma) in the ocular adnexa. However, the pathogenetical significance of the infection has not been fully elucidated. Many previous studies have shown controversial results regarding *C.* *psittaci* detection rates in said patients, ranging from 0 to 87%. We investigated the presence of *C. psittaci* in a single institutional cohort (*n* = 150) of ocular adnexal MALT lymphoma (OAML) patients in Korea. We tried to exclude the methodological biases derived from the different primer sets in polymerase chain reaction-based studies. For that reason, we applied five sets of primers, including four previously reported and one newly designed primer set. There was no case of *C. psittaci*-positive OAML in repeated trials validated with appropriate positive and negative controls. All 150 cases showed negative results with five primer sets. These results suggest that the pathogenetic role of *C. psittaci* in ocular adnexal MALT lymphoma might have been overestimated to date, at least in the Korean population. Therefore, the molecular diagnosis of *C. psittaci* is considered a very low priority.

## 1. Introduction

Extranodal marginal zone lymphoma of mucosa-associated lymphoid tissue (MALT lymphoma) is a low-grade lymphoma that originates in heterogeneous marginal zone small B cells. In the ocular adnexa, MALT lymphoma involves conjunctiva-associated lymphoid tissue, and it is the most common type of lymphoma in this organ. The incidence of ocular adnexal MALT lymphoma (OAML) is continuously increasing; the annual increase is more than 6% [1]. Unlike other types of lymphoma, MALT lymphomas are caused by prolonged chronic inflammation that results from infection, autoimmunity, or other unknown stimuli. It is widely known that a predominant association of gastric MALT lymphoma with *Helicobacter pylori* infection exists. Therefore, *Helicobacter pylori* is considered to be a major causative infective factor.

Since the initial discovery, several reports about *Chlamydophila psittaci* (*C. psittaci*) infection in ocular adnexal MALT lymphoma (OAML) patients have been published worldwide [2,3]. *C. psittaci* is a small bacterium that is transmitted by birds and mostly causes *C. psittaci* pneumonia. *C psittaci* can affect organ systems other than the respiratory tract, resulting in endocarditis, myocarditis, hepatitis, arthritis, keratoconjunctivitis, and encephalitis. Initially, an Italian group reported that they had detected a chlamydia infection rate of about 80% in forty OAML cases [2]. However, the detection rate is quite variable, ranging from 0 to 87% across several reports [4]. Moreover, the pathogenic significance of *C. psittaci* infection has not been fully elucidated. Therefore, controversy about the role of *C. psittaci* in lymphomagenesis remains.

Given this controversy, we questioned whether *C. psittaci* is a crucial pathoetiological factor in MALT lymphoma in the Korean population. The aim of this study was to verify the prevalence of *C. psittaci* infection in OAML using broad-spectrum primer sets and to determine its pathoetiological significance.

## 2. Materials and Methods

### 2.1. Patient and Tissue Samples

We collected archival biopsy or excision specimens from patients diagnosed with ocular adnexal extranodal marginal zone lymphoma of mucosa-associated lymphoid tissue (MALT lymphoma) between 2008 and 2014 at Seoul St. Mary’s Hospital. OAML is defined as lymphoma in the ocular adnexa, including conjunctiva, lacrimal gland, orbital fat, eyelid, and lacrimal sac. At the time of the diagnosis, immunohistochemical stains, including CD20, CD3, CD79a, Bcl2, Bcl6, pancytokeratin, cyclin D1, and Ki67, were routinely performed for the accurate diagnosis. Lymphoid proliferative tumors that displayed marked monocytoid marginal zone B-cell expansion, often with Bcl2 expression and low Ki67 index, were diagnosed as MALT lymphoma. One case with equivocal histology was further studied with an IgH gene rearrangement test and showed monoclonality. The original hematoxylin and eosin stained sections were reviewed by two expert pathologists (G.P. and H.L.). Diagnoses were confirmed by histological review, and cases were excluded for the following reasons: (1) a lack of a sufficient amount of tumor tissue for the extraction of DNA, (2) transformation into high-grade lymphoma, (3) discordance between the two expert pathologists on the diagnosis or uncertainty in the diagnosis. Finally, 150 MALT lymphoma cases were selected for the study. Patient demographics and clinical data were acquired from hospital medical records. This study was approved by the Institutional Review Board of Seoul St. Mary’s Hospital (KC21ISIS0190).

### 2.2. Molecular Detection of C. psittaci

For *Chlamydophila* detection, DNA was extracted from two 10 μm sections of formalin-fixed, paraffin-embedded tissue blocks. The representative MALT lymphoma lesions were marked and manually microdissected under a stereomicroscope. DNA extraction was performed using the Maxwell16^®^ FFPE plus LEV DNA purification kit (Promega, Leiden, The Netherlands) according to manufacturer′s instructions. The purified DNA was measured with a NanoDrop (Thermo Scientific, Rockford, IL, USA). With the purified DNA, polymerase chain reaction (PCR) tests were performed using the GeneAmp^®^ PCR System 9700 (Applied Biosystems, Carlsbad, CA, USA), and double-checking was performed using MCE^®^-202 MultiNA (Shimadzu Corporation, Kyoto, Japan). Four primer sets for PCR and one pair of primer sets for nested PCR were used in this study to perform a chlamydia-specific PCR analysis (Table 1). We prepared four sequence sets with reference to previous publications, resulting in both positive and negative results [2,5,6,7]. In addition, the fifth set of oligonucleotides targeting 16S gene was newly designed for this study to enhance the performance of the test (Table 1). All five primer sets were carefully verified using the *beta-globin* gene and positive control DNA. Well-qualified DNA samples of *C. psittaci* were acquired from the Korea Centers for Disease Control and Prevention (KCDCP) and used as positive controls. DNA samples extracted from reactive inguinal nodal tissues were used as negative controls. All the PCRs were performed with a volume of 20 μL containing 100 ng of template genomic DNA, 10× Taq buffer, 2.0 mM of MgCl_2_, 200 µM of dNTPs, 0.2 µM of each primer, and 0.5 unit of HotStar Taq DNA polymerase (Qiagen Inc, Valencia, CA, USA). The cycling protocol was carried out as follows: 1 cycle of 95 °C for 5 min; then, 40 cycles consisting of denaturation for 30 s at 94 °C, annealing for 30 s, and elongation for 30 s at 72 °C; and finally, 72 °C for 5 min. For annealing, the temperature of 55 °C was used for primers 1, 3, 4, and 5; and the temperature of 60 °C was used for primer 2. PCR products were separated by electrophoresis in 2% agarose gels with 0.5× Tris-acetate-EDTA buffer, visualized with ethidium bromide, and photographed.

## 3. Results

A total of 150 patients with a median age of 39 years (range, 24–63) were included in this study. There was a female predominance (2.5:1), and most of the patients were classified as stage I (94%). In addition, the majority of our patients lived in non-rural areas (85%) (Table 2).

**Figure 1:**
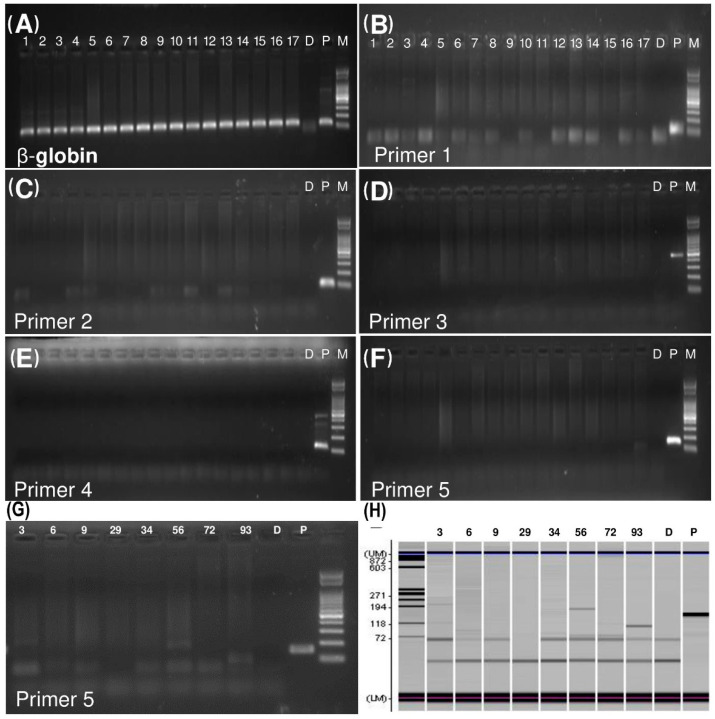
*C. psittaci* detection by polymerase chain reaction (PCR) in ocular adnexal MALT lymphoma tissues. (**A**) The DNA quality of the samples was adequate, as shown by clear beta-globin bands. (**B**–**F**) There is a clear single band in each positive control column, but there is no other amplified PCR product in the other tissue sample columns. (**G**) In the initial PCR test, a few samples showed ambiguous bands near the level of the positive control DNA. (**H**) The ambiguous bands were confirmed negative when observed using a high-resolution device. (D, distilled water as a negative control; P, positive control; M, DNA size standard ladder).

*Beta-globin* showed distinct DNA bands, indicating successful genomic extraction, and the quality of the DNA in all the samples was sufficient to generate products at least 100 bp in size (Figure 1A). We observed the PCR products of the control DNA that were amplified using primers 1, 2, 3, 4, and 5 (primer 1—111 bp PCR product, primer 2—100 bp PCR product, primer 3—430 bp PCR product, primer 4—125 bp PCR product, primer 5—149 bp PCR product) (Figure 1B–F). However, we did not detect clear evidence of *C. psittaci* DNA in any MALT lymphoma samples by using five different sets of primers in PCR. Distinct DNA bands at the positive control DNA band level were not observed (Figure 1B–F). Several samples (samples 3, 6, 9, 29, 34, 56, 72, and 93) showed ambiguous, faint bands at the positive control DNA band level. We re-amplified these samples and evaluated the samples using a high-resolution MultiNa PCR device, and these findings were confirmed to be negative (Figure 1G,H). The experiments were performed once for all the samples.

## 4. Discussion

MALT lymphoma is a well-demonstrated model of antigen-driven malignancy. Many other lymphoma entities have also been found to be related to infectious agents, but the idea that chronic inflammation due to infection drives the lymphocyte transformation and development of MALT lymphoma has been thoroughly investigated. Since the link between *Helicobacter pylori* and *H. helmenii* in gastric MALT lymphoma was first discovered, *Campylobacter jejuni* in small intestinal lymphoma and *Borrelia burdorferi* in cutaneous lymphoma have also been discovered [8].

In ocular adnexa, OAML has been described in the context of chronic conjunctivitis, which can be associated with *Chlamydia* infection [9]. Ferreri et al. found *C. psittaci* infection in 80% (32/40) of the DNA samples of a group of European patients affected by OAML [2]. They further demonstrated the efficacy of bacteria-eradicating therapy with doxycycline and showed an outstanding response. In their study, a group of 27 patients affected by OAML—11 *C. psittaci* DNA-positive and 16 *C. psittaci* DNA-negative—was treated with doxycycline. A total of 48% (13/27) of the patients, both *C. psittaci* DNA-positive and negative patients, responded to the treatment. The researchers postulated the doxycycline’s removal of *C. psittaci* cured the lymphoma [10]. A recent clinical trial using doxycycline also proved bacteria-eradicating therapy to be an effective and active treatment for stage I *C. psittaci*-positive OAML [11]. Moreover, isolated *C. psittaci* from biological samples of the lymphoma patients was found to be viable and still infectious [12]. The aforementioned evidence supported that *C. psittaci* is a causative pathogen in a subset of patients with OAML. The association of *C. psittaci* infection and OAML was supported in other studies that detected *C. psittaci* DNA in OAML cases. The detection rates, however, were highly variable—from 78% (26/33) in a study from Korea [3] to 0% in studies from the USA and Japan [5,6]. A multi-national study demonstrated the presence of *C. psittaci* DNA in OAML tissues from Germany (47%), the east coast of the United States (35%), the Netherlands (29%), Italy (17%), the United Kingdom (12%), and southern China (11%) [13]. Several studies conducted in the USA, Japan, China, and the Netherlands reported no evidence of *C. psittaci* infection in their patients with OAML [14].

There are several hypotheses regarding the cause of the variable association of *C. psittaci* and OAML. This variation may be related to the differences in the modality and sensitivity of the detection methods used in different studies. Another hypothesis is that the genetic background of different populations and other epidemiological risk factors in different geographic areas may affect the lymphomagenesis. Carugi et al. analyzed the association of *C. psittaci* with the OAML of African patients and Italian patients. They found no association with *C. psittaci* in the African patients but confirmed an association in Italian patients [15]. They showed that geographic and environmental conditions might affect the incidence of lymphomas. However, great variations in the infection rate even in the same geographical areas still raised whether *C. psittaci* is a universal pathogen causing OAML.

Korea is considered a region with a high prevalence of *C. psittaci* infection in OAML cases. In a total of 78 cases, *C. psittaci* DNA was found in 50% to 78% [3,16,17]. Our study did not find any association between *C. psittaci* and OAML in Korean patients, which contrasts with the results of previous studies. We performed a rigorous analysis with the largest cohort and used broad-spectrum primer sets to reduce the variability caused by the sensitivity of the designed primers. The reason for the discrepancy between our results and those of others is unclear. We observed in the data of this study that it was neither a genetic nor geographic variable that caused the difference in *C. psittaci* infection in OAML. Besides the genetic background of the regional populations, other epidemiological risk factors, such as living conditions, may affect the lymphomagenesis. Many of the subjects with *C. psittaci* infection lived in rural areas and had prolonged contact with household animals [2]. In our case, most of the patients lived in urban areas, unlike the Italian studies [2,18]. It is hard to compare living conditions/localities between our study and the previous Korean studies, because relevant data were not provided in those studies.

We should be cautious in deciding a treatment option based on *C. psittaci* as a causative agent in OAML patients, even in regions where high rates of infection, such as Korea, have been reported. We note that *C. psittaci* infection in individual patients could vary widely, as our Korean cohort showed the absence of infection.

Many previous studies have used multiplex touchdown enzyme time-release PCR (TERT-PCR), as described by Madico et al. [7], and CPS 100 and CPS 101 primers for *C. psittaci* detection. A limitation of our study is that we did not use the TERT-PCR method. Although the PCR method and primers—except for homemade primer set 5—used in this study were selected from the published literature, the sensitivity and specificity of the different detection methods could have affected, at least partially, the study results. However, the quality of the DNA extracts prepared from the tissue samples and the products of different PCR primers was high, and demonstrated the suitability of the detection method.

Korea is considered a region with a high prevalence of *C. psittaci* infection in lymphoma, like Italy. In conclusion, our study first showed a low prevalence of *C. psittaci* infection in OAML in Korea. The present study is especially meaningful in that it analyzed the largest cohort to date concerning the association of *C. psittaci* and OAML. Our data suggest the possibility that the pathogenetic role of *C. psittaci* in OAML may have been overestimated so far, at least in the Korean population. Therefore, the need for the detection of *C. psittaci* for the diagnosis and treatment of OAML seems low based on our data. Moreover, physicians should carefully apply the antibiotic treatment strategy for the OAML in regions with low *C. psittaci* infection prevalence.

## Figures and Tables

**Table 1:** Primer sets for polymerase chain reaction detection of *Chlamydophila psittaci*.

Primer No.	Title 2	Amplicon Size (bp)	Reference
Primer 1 (CPS 100/101)	Forward: 5′-CCC AAG GTG AGG CTG ATG AC-3′ Reverse: 5′-CAA ACC GTC CTA AGA CAG TTA-3′	111	Madico et al. [7]
Primer 2	Forward: 5′-ATA CAG GGT GAT AGT CCC GTA GAC-3′ Reverse: 5′-GTG GTC TCC CCA GAT TCA GAC TAG-3′	100	Ferreri et al. [2]
Primer 3 (1st step primer for nested PCR)	Forward: 5′-ACG GAA TAA TGA CTT CGG-3′ Reverse: 5′-TAC CTG GTA CGC TCA ATT-3′	430	Vargas et al. [5]
Primer 4 (2nd step primer for nested PCR)	Forward: 5′-ATA ATG ACT TCG GTT CTT ATT-3′ Reverse: 5′-TGT TTT AGA TGC CTA AAC AT-3′	127	Daibata et al. [6]
Primer 5 (Newly designed primers)	Forward: 5′-CGT TGA CTC AAC CTG CAA AG-3′ Reverse: 5′-CAA CCT AGT CAA ACC GTC CT-3′	149	-

**Table 2:** Characteristics of ocular adnexal MALT lymphoma patients.

Characteristics	Number of Patients (*n* = 150)	Percentage
Age, years		
Median (range)	39 (24–63)	
Sex		
Female	107	71%
Male	43	29%
Site of tumor		
Conjunctiva	125	83%
Lacrimal gland	0	0%
Eyelids	6	4%
Lacrimal drainage apparatus	12	8%
Other orbital tissues	7	5%
Laterality		
Unilateral	104	69%
Bilateral	46	31%
Residency in rural areas	23	15%
TNM stage		
Stage I	141	94%
Stage II-III	9	6%
Treatment		
RT	118	79%
CVP	15	10%
RT + CVP	7	5%
No additional treatment	10	7%

CVP, cyclophosphamide, vincristine, and prednisone combination chemotherapy; RT, radiation therapy.
